# Recent advances and challenges in the recovery and purification of cellular exosomes

**DOI:** 10.1002/elps.201800526

**Published:** 2019-08-12

**Authors:** Sergio Ayala‐Mar, Javier Donoso‐Quezada, Roberto C. Gallo‐Villanueva, Victor H. Perez‐Gonzalez, José González‐Valdez

**Affiliations:** ^1^ Tecnologico de Monterrey School of Engineering and Science, Av Eugenio Garza Sada 2501 Sur Monterrey NL Mexico

**Keywords:** Bioseparations, Cellular vesicles, Downstream processing, Exosomes, Exosome purification

## Abstract

Exosomes are nanovesicles secreted by most cellular types that carry important biochemical compounds throughout the body with different purposes, playing a preponderant role in cellular communication. Because of their structure, physicochemical properties and stability, recent studies are focusing in their use as nanocarriers for different therapeutic compounds for the treatment of different diseases ranging from cancer to Parkinson's disease. However, current bioseparation protocols and methodologies are selected based on the final exosome application or intended use and present both advantages and disadvantages when compared among them. In this context, this review aims to present the most important technologies available for exosome isolation while discussing their advantages and disadvantages and the possibilities of being combined with other strategies. This is critical since the development of novel exosome‐based therapeutic strategies will be constrained to the effectiveness and yield of the selected downstream purification methodologies for which a thorough understanding of the available technological resources is needed.

**Abbreviation**
HIC: hydrophobic interaction chromatography

## 1 Introduction

Living organisms have evolved to use different highly‐selective intercellular communication pathways that allow the transport of biological signals and materials, which guarantee the correct function of cells, tissues, organs, and systems 1. In this context, a particular type of cell‐derived vesicles, exosomes, were first observed in reticulocyte as internal bodies that were released from the cell as endosomal vesicles merged with the cell membrane. In this first observation, exosomes were found to play an important role in the removal of plasma membrane proteins 2. However, since exosomes can be virtually obtained from all types of eukaryotic cells (but most commonly in those of the immune system) they have been associated with the transport of nucleic acids, proteic, and other signaling cargos in different models 3. Recent evidence suggests a crucial role of exosomes in many aspects of disease from metastasis in cancer, to cardiomyocyte size in heart failure 4, 5, 6. The study of these vesicles is paramount to fully understand the physiopathology of the leading causes of death worldwide. Moreover, previous studies have suggested the potential use of exosomes as biomarkers in cancer, cardiovascular disease, and neurodegenerative disorders 7, 8, 9, 10.

As naturally occurring nanocarriers, the use of exosomes in therapeutic strategies have also been explored. Drugs and many other compounds may be loaded into them, harnessing highly specific delivery systems 8, 11. Furthermore, functionalization techniques have investigated the use of exosomes as modulators of physiopathological processes 12, 13. For instance, these vesicles have been an important aid in the development of recent cancer immunization strategies 14 showing an enormous potential in different health‐related applications. However, the use of exosomes as therapeutic agents is still far away from becoming a reality since their procurement is usually a difficult, time‐consuming, and low‐yield task.

Structurally, exosomes are composed by a lipid bilayer membrane with specific surface proteins, which differ between cell types although they possess a common set of conserved protein molecules 15. Exosomes are usually smaller than other extracellular vesicles (between 15 and 75 nm) and thus require special considerations in their separation and analysis 16. Although different approaches for the separation, purification, and analysis of exosomes have been explored, so far, there is no methodology providing enough robustness regarding purification yield, selectivity, and reproducibility. In fact, because of the inherent biochemical properties of these vesicles and the enormous differences between them that depend on the matrix from which they are obtained, usually a combination of techniques needs to be tailored to achieve the desired purification outcomes. In this sense, different physical and/or chemical methodologies have been studied and proposed to achieve a good exosome purification yield. This work aims to establish the current state of the art in exosome isolation strategies highlighting the advantages and disadvantages of each of the most commonly used techniques while presenting a perspective of the future of this important topic. Table 1 17, 18, 19, 20, 21, 22, 23, 24, 25, 26, 27, 28, 29, 30, 31, 32, 33, 34, 35, 36, 37, 38, 39, 40, 41, 42, 43, 44, 45, 46, 47, 48, 49, 50, 51, 52, 53, 54 presents an overview of the different types of exosomes regarding their biological source, their functions, applications and isolation procedures currently used to procure them.

**Table 1:** Exosome classification based on the producer cell type

Source	Function	Application	Isolation	References
***Nervous system***				
Neuron derived exosomes Schwan‐Cell derived exosomes Microglia‐Cell derived exosomes Astrocyte derived exosomes Oligodendrocyte derived exosomes	Reciprocal control of excitatory synapse, modulation of axonal branching, neuronal activity and plasticity, release and trans‐synaptic transmission of proteins	Spinal cord and peripheral nerve regeneration, targeted therapy for neurological disorders, early detection of neurodevelopmental disorders, neurodegenerative disorders, and traumatic brain injury	Ultracentrifugation of neural cell culture medium, differential ultracentrifugation of brain tissue homogenates, and precipitation of cerebrospinal fluid	17, 18, 19, 20, 21
***Cardiovascular system***				
Cardiomyocyte derived exosomes Cardiac Telocyte‐derived exosomes Cardiac Progenitor‐Cell derived exosomes Endothelial‐Cell derived exosomes Cardiosphere‐derived exosomes	Induction of cardiomyocyte hypertrophy, regulation of oxidative stress and inflammation, inhibition of apoptosis, induction of cardiac endothelial cell proliferation, and modulation of cardiac fibrosis	Biomarker of myocardial injury, targeted therapy for myocardial infarction and heart failure, and prognostic marker of cardiovascular diseases	Differential ultracentrifugation of cardiomyocyte homogenates, multi‐step centrifugation of cardiac fibroblast culture medium, and precipitation of pericardial fluid	6, 22, 23, 24
***Liver***				
Hepatocyte derived exosomes Cholangiocyte derived exosomes Kupffer Cell‐derived exosomes Liver Endothelial Cell‐derived exosomes Hepatic Stellate Cell‐derived exosomes	Induction of hepatocyte survival, growth, migration and proliferation, trans‐differentiation of myofibroblast hepatic stellate cells, regulation of fibrosis, and the inflammatory response in the liver	Targeted therapy for liver regeneration, biomarkers for assessing the safety of liver transplantation, diagnostic and prognostic markers of nonalcoholic fatty liver disease, and alcoholic liver disease	Density‐gradient ultracentrifugation of liver tissue homogenates, differential ultracentrifugation, and precipitation of primary hepatocyte culture medium	25, 26, 27, 28, 29
***Stem cells***				
Mesenchymal Stem Cell‐derived exosomes Neural Stem Cell‐derived exosomes Induced Pluripotent Stem Cell‐derived exosomes Embryonic Stem Cell‐derived exosomes Endothelial Progenitor Cell‐derived exosomes	Development and growth of the embryo, adult tissue regeneration, differentiation and transformation, immunomodulation, and stromal remodeling	Induction of tissue remodeling, targeted regenerative therapy for neoplasms, ischemic myocardium, graft‐versus‐host disease, memory dysfunction, autoimmune disorders, and other degenerative disorders	Differential ultracentrifugation and one‐step sucrose cushion ultracentrifugation of stem cell culture medium, and precipitation from stem cell culture medium	30, 31, 32, 33
***Skeletal muscle***				
Myotube derived exosomes Muscle‐derived Fibroblasts exosomes	Control of myoblast differentiation and proliferation, regulation of skeletal muscle metabolic homeostasis, oxidative stress and inflammation	Targeted therapy for muscular dystrophy, insulin resistance and other metabolic disorders affecting muscle physiology	Differential ultracentrifugation of myoblast and myotube cell culture medium	34, 35
***Pancreas***				
Pancreatic stellate cell‐derived exosomes β‐cell‐derived exosomes	Regulation of cell proliferation, migration and modulation of immune responses (regulation of chemokines expression in pancreatic cells, antigen deliver and activation of dendritic cells)	Proposed as biomarkers for pancreatic cancer and as therapeutic target to control autoimmune responses in type‐1 diabetes	Differential ultracentrifugation of cell culture medium	36, 37, 38
***Urinary exosomes***				
Bladder‐derived exosomes Kidney‐derived exosomes Prostate‐derived exosomes	Induction of cell migration, modulation of angiogenesis, control of biological processes associated to the progression of advanced fibrotic disease, and regulation of inflammatory processes	The exosome concentration and several exosomal miRNAs and proteins are proposed as biomarkers for bladder cancer, diabetic nephropathy, lupus nephritis, Parkinson's disease, IgA nephropathy, and prostate cancer	Differential ultracentrifugation and precipitation of human urine	39, 40, 41, 42, 43
***Immune system***				
Dendritic cell‐derived exosomes B‐cell‐derived exosomes Macrophage‐derived exosomes T‐cell derived exosomes	Antigens distribution and delivery for activation of immune response, elimination of immune cells for suppression of immune response, and regulation of the expression of pro‐inflammatory molecules	Potential use as nanocarriers for the delivery of immunomodulatory molecules, and vaccines for immune therapy	Differential ultracentrifugation and ultracentrifugation followed by sucrose gradient purification of culture medium	44, 45, 46, 47, 48
***Breast***				
Breast milk‐derived exosomes	Regulation of immune response, protective role against vertical transmission of HIV‐1, and stimulation of intestinal stem cell activity	Prevention of necrotizing enterocolitis in intolerant breastfeeding infants and immune therapy	Differential centrifugation of human breast milk of healthy mothers and precipitation of human and rat breast milk	49, 50, 51, 52
***Tumor‐derived exosomes***				
Glioblastoma cell‐derived exosomes Colon cancer cell‐derived exosomes Melanoma cell‐derived exosomes Nasopharyngeal carcinoma cell‐derived exosomes Prostate cancer cell‐derived exosomes Cervical cancer cell‐derived exosomes Diffuse large B‐cell lymphoma side population cell‐derived exosomes Lung cancer cell‐derived exosomes Bladder cancer cell‐derived exosomes Breast cancer‐cell derived exosomes Mesothelioma cancer cell‐derived exosomes Pancreatic cancer cell‐derived exosomes Renal cancer cell‐derived exosomes Chronic myeloid leukemia cell‐derived exosomes Hepatocellular carcinoma cell‐derived exosomes	Promotion of tumor proliferation, invasion and metastasis, enhance angiogenesis, impair immune response and increase cancer resistance to therapies	Proposed as biomarkers for the detection of several types of cancer and nanocarriers for targeted drug delivery against cancer proliferation	Differential centrifugation and precipitation of human fluids and cell culture medium	53, 54

## 2 Exosome recovery and purification

Regardless of their final intended use, exosomes need to be isolated from varied and complex biological samples. This is a critical procedure, since exosomes must preserve their physicochemical properties and biological function after isolation. Furthermore, many challenges arise when isolating these naturally‐occurring particles. For example, exosomes belong to a large domain of extracellular vesicles, some of which present overlapping physicochemical properties 55. Moreover, exosomes themselves exhibit high heterogeneity in size, cargo, and surface markers 15, 56, 57. Therefore, exosome isolation methods must be efficient, specific, flexible, and have long‐term perspective of clinical applications. In this context, current isolation methods include ultracentrifugation, filtration, precipitation, chromatography, immunoaffinity capture and microfluidics, which will be discussed in the following subsections. A description of the different and most commonly used exosome isolation techniques, as well as some of the recommended applications for each one is presented in Table 2.

**Table 2:** Proposed classification of exosome isolation methods based on their main principle. Advantages and disadvantages are highlighted after each method. Grouped rows indicate the most commonly associated techniques. Feasibility of the different potential applications depending on the isolation method are also identified

				Applications			
Method	Type	Advantages	Disadvantages	Nucleic acid quantification and sequencing	Biomarker screening	Protein quantification and identification	Drug delivery systems
Centrifugation	Physical	Simple protocols Preservation of physicochemical properties	Low purity Specialized equipment required	✓	✓	✗	✓
Ultrafiltration	Physical	Fast protocols Cheap materials High protein and RNA yield	Low purity Exosome deformation and extrusion	✓	✓	✗	✗
Precipitation	Physicochemical	Highly reproducible High vesicle and RNA yields Requires only lab‐bench equipment	Low purity Exosome aggregation	✓	✓	✗	✗
Chromatography	Physicochemical	High exosome purity yields Preservation of physicochemical properties	Medium to high processing times Exosome degradation due to selected operation buffers	✓	✓	✓	✓
Microfluidics	Physical	Miniaturization Device functionalization Short analysis times	Low reproducibility Low exosomal yields Exosome aggregation	✓	✓	✓	✓
Immunocapture	Chemical	High specificity High purity Potential scalability	Expensive materials Low exosomal and RNA yields	✓	✗	✓	✗

### 2.1 Ultracentrifugation

The current golden standard for exosome isolation is ultracentrifugation 58. As known, this technique exploits the particle movement principle due to gravitational acceleration in an inertial field 59. Differential and density gradient ultracentrifugation are among the most commonly used ultracentrifugation methods for exosome isolation 60.

Differential ultracentrifugation is commonly known as the pelleting method, because it allows to obtain pellets containing exosomes. It is also referred to as the simple ultracentrifugation method since it only requires several ultracentrifugation steps 61. To date, this is the most widely used method for isolating exosomes and has been successfully used in a variety of biological fluids and cell culture media 62, 63. During differential ultracentrifugation, exosomes are separated based on their density and size. Thus, contamination from other vesicles, molecules or particles that overlap in these parameters is expected. To reduce the presence of cell debris and large vesicles, cleaning steps are needed before pelleting the exosomes 64, 65, 66. In this context, differential centrifugation at low gravitational forces has shown to remove such contaminants, and it is widely recommended. In each cleaning step, contaminants are precipitated, and the final supernatant is finally ultracentrifuged to pellet the exosomes 67.

Exosomes are sedimented by ultracentrifugation using forces between 100 000–200 000 × *g* for 1 to 2 h 23, 66, 68. The recovered pellet is then resuspended in a convenient buffer such as PBS. Sometimes further purification steps may be useful for certain types of downstream exosome analysis 69. However, these need to be adjusted depending on the requirements of such analytical methods, since further purification steps will also decrease the final exosome recovery yield.

Multiple parameters can alter the consistency of a differential ultracentrifugation protocol 70. Different rotor types require specific centrifugation parameters to achieve exosome sedimentation, not only fixed angle and swinging bucket rotors have different sedimentation pathlengths but also the *g*‐force differs according to the distance from the rotational axis 68. Thus, the centrifugation time and *g*‐force should be properly adjusted to fit the particular rotor used. In this regard, the *k*‐factor can be used to perform proper rotor conversion calculations. Cvjetkovic et al. compared, adjusted, and unadjusted protocols using fixed‐angle and swinging‐bucket rotors and found that an unadjusted protocol results in a lower exosomal RNA yield 70. According to these results, a specific *g*‐force does not pellet exosomes with the same efficiency in different types of rotors, and the centrifugation time needs to be properly considered. Otherwise, sample purity, protein content and exosomal yield will be compromised 68. Nonetheless, differential ultracentrifugation has been used to isolate exosomes from cell culture medium, urine, plasma, cerebrospinal fluid, among other biological matrices 71, 72, 73, 74.

It should be noted that the *g*‐force used during ultracentrifugation protocols has a significant effect on the purity and yield of exosomes 75. Moreover, exosome sedimentation efficiency has been found to vary among cell lines. Jeppesen et al. demonstrated a significant difference in the sedimentation profile of vesicles and proteins from FL3 cells compared to HEK293 cells 76.

Using the particle to protein ratio as a measure of sample purity, Jeppesen et al. showed that the samples with the highest purity are found after ultracentrifugation at 67 000 × *g* for KEK293 and after 100 000 × *g* for FL3. Remarkably, different expression patterns for exosomal markers TSG101 and syntenin were also found in the previous study, suggesting that TSG101 may be more expressed on larger or denser exosomes that sediment more easily, and that syntenin may be more expressed on smaller or less dense exosomes that sediment at higher *g*‐forces. These observations highlight the importance of adapting ultracentrifugation protocols considering the cell line, since it is also possible that exosomes from different origins exhibit different sizes 76.

The exosomes isolated with this method have been successfully identified by immunoblotting, electron microscopy, and quantified with nanoparticle tracking 77, 78, 79. For instance, exosomes have been purified from culture medium of cardiac fibroblasts by a simple differential ultracentrifugation protocol. These fibroblast‐derived exosomes were confirmed by the presence of CD63 using Western Blot and flow cytometry. Moreover, exosome uptake experiments were performed with the PKH26 fluorescent dye and RNA sequencing was performed, showing that cardiac fibroblasts secrete exosomes to mediate cardiomyocyte hypertrophy 23.

In general terms, differential centrifugation methods are considered easy to perform, moderately time‐consuming and do not require sample pretreatments 58. However, as it has been mentioned, this method is not specific and contamination with other extracellular vesicles is unavoidable. If the protocol is not well standardized and adapted (in terms of time and gravitational force) to the characteristics of the equipment being used, exosome isolation will not be consistent, and losses will occur inevitably 59.

On the other hand, density gradient centrifugation is a well‐known technique for the purification of cellular organelles 80, 81, 82. If exosomes are considered as such, this ultracentrifugation variation is well within the capabilities of performing successful exosome isolations. In this technique, a continuous or stepwise density gradient is formed in the medium during (or before) the centrifugation procedure 58, 68. Thus, once submitted to the centrifugal force, exosomes will move through the gradient according to their density 83.

Density gradient ultracentrifugation results in a density region of interest, where exosomes are concentrated 84. Density gradient ultracentrifugation can be performed by the bottom‐ or the top‐loading method. In the case of bottom‐loading method, also called isopycnic ultracentrifugation the major effector is density 85. On the other hand, in the top‐loading method, also called moving‐zone ultracentrifugation, the major effector is particle size 84. Exosomes may move to a zone where they have the same density as the medium, as is the case for isopycnic ultracentrifugation in which a density gradient medium embracing the entire range of solute densities is used 85, 86. Exosomal density has been reported between 1.10 and 1.21 g/mL in cesium chloride density gradients 55. On its part, moving‐zone ultracentrifugation uses a gradient density medium with a lower density than the sample 84. In this particular variation, exosomes and all the other solutes will eventually precipitate, thus the centrifugation time dictates the position of the vesicles according to their size. A single or double sucrose cushion is often used in both techniques to avoid losses and excessive contamination 87, 88. Regardless of the density gradient ultracentrifugation procedure being used, the fraction of interest is subjected to another round of simple ultracentrifugation at >100 000 × *g* for further purification 83. It should be noted that this particular technique may be considered complex, time‐consuming and inefficient, due to considerable exosome loss 68. However, density gradient centrifugation may be more specific than differential ultracentrifugation to certain exosome types, especially when using moving‐zone ultracentrifugation.

As an example, density gradient ultracentrifugation has been successfully used to isolate exosomes from culture supernatants of N2a cells for the study of Alzheimer's disease 89. In this case, differential ultracentrifugation was first used to pellet the exosomes. Then, the exosomes were resuspended in a 2.5 M sucrose solution, and a step gradient of sucrose was layered over it. The gradient was further spun, and fractions were collected for immunoblotting and electron microscopy. This study showed exosomal proteins bound to Alzheimer's disease signature plaques, suggesting a role in the pathogenesis of such disease.

Another approach to avoid contamination and excessive losses is to use an iodixanol cushion or gradient. A recent study showed that differential centrifugation, ultracentrifugation with an iodixanol cushion, and ultracentrifugation on an iodixanol density gradient yielded the same number of particles 90. This was the same result as for clearance from an *in vivo* model. Nonetheless, according to electron microscopy, the exosomes of the gradient method were more dispersed.

It should be noted that another advantage of ultracentrifugation protocols is their capability to clearly yield vesicles with diameters of approximately 100 nm, which are observable by electron microscopy 66. Other methods, such as precipitation, usually result in large aggregates and non‐spherical lumps with diameters larger than 1 µm 58. Moreover, exosome size distribution and zeta potential appear to be unchanged by ultracentrifugation 90, which is of great importance for the different exosome applications that are being proposed and studied.

### 2.2 Filtration‐based strategies

Ultrafiltration is another available technique to obtain exosome enriched samples from a variety of sources including cell culture medium and biological fluids 91. In this process, extracellular vesicles suspended in a solution can be separated by size or molecular weight. Usually, different forces are applied to make them pass through (or be retained on) a selective membrane. Centrifugal force, pressure or vacuum are usually applied for ultrafiltration through a membrane that is commonly built from low protein affinity materials. In this context, nanomembranes have been used to enrich exosome samples as effectively as ultracentrifugation, in smaller volumes and shorter times 58, 68.

Ultrafiltration is usually a complement to other exosome isolation techniques 92. Several protocols use this method as a cleaning step, especially after ultracentrifugation and precipitation techniques 93, 94, 95. Such protocols have demonstrated to selectively separate exosomes as confirmed by cryo‐electron microscopy, dynamic light scattering, and Western Blot analysis for CD63 and CD81 96. However, ultrafiltration with selective filtration membranes can also be used to isolate exosomes by itself. Several protocols, which are faster, easier and less expensive than ultracentrifugation, are available 68. Filtration by size and molecular weight are usually coupled in the same protocol 97. Filters with pore sizes of 0.8, 0.45, and 0.22 µm and membranes with a molecular weight cut‐off ranging from 10 to 100 kDa are commonly used 14, 61, 98, 99, 100, 101. Furthermore, exosome‐derived RNA and protein concentrations are considerably larger than in most methods, as the final sample will be highly concentrated 102. For instance, this method has been used to obtain highly‐concentrated urinary exosomes that may serve as biomarkers of renal diseases 87, 103. First, raw urine samples were clarified using conventional centrifugation. Then, the clarified urine was microfiltered through a 0.1 µm pore size filter and further concentrated using a 10 kDa molecular weight cut‐off membrane. The presence of exosomes was validated by electron microscopy.

Nevertheless, ultrafiltration, a simple protocol, is incapable of isolating only exosomes, as microvesicles and apoptotic bodies will also be present in the resulting product. Moreover, large amounts of highly abundant proteins, that mimic exosome size or molecular weight, will also be found in the resulting solution. Such contamination arises from the physical limits of the procedure and the overlapping properties of the particles in the matrix being processed. Furthermore, the effects of the applied force and the contact with the membrane on the exosomes need to be further studied. Potential deformation and exosome losses due to extrusion and membrane binding are expected 104. However, it has also been reported that exosomes isolated by this method have physical properties comparable to those obtained by ultracentrifugation or precipitation 90.

Other filtration techniques include hydrostatic filtration dialysis and size‐exclusion chromatography. In hydrostatic filtration dialysis, the sample moves through a dialysis membrane and exosomes are retained as the solvent and other solutes equilibrate 105. This method is particularly useful for enriching and concentrating the sample. It shows one of the highest exosomal yields of all methods, and does not require ultracentrifugation 92, 106. It is also scalable and applicable to a large volume 107.

Size‐exclusion chromatography, on its part, is a filtration procedure in which a stationary phase sorts dissolved particles according to their size distribution. Depending on their hydrodynamic radii, these particles will be differentially eluted from the column 108. This technique is hyphenated with other methods like ultracentrifugation, immunoaffinity capture, and even precipitation since these combinations usually enrich the sample and produce better quality exosomes (based on morphology evaluation by electron microscopy) 109, 110. A recent study compared exosome isolation by size‐exclusion chromatography to polymer‐based precipitation and protein organic solvent precipitation 111. Although the three methods successfully isolated exosomes (confirmed by flow cytometry and Western Blot of CD9, CD63, and CD81) only size‐exclusion chromatography did not alter vesicular size and produced the highest purity as observed by cryo‐electron microscopy. This procedure preserved biological function but more importantly, it was found that size‐exclusion chromatography usually yields the highest representation of exosomal biomarkers 112. Limitations found include the sample volume (as it depends on the column volume) and its yield (as it is lower than other techniques when used on its own) 113. It is important to mention that other chromatographic modes, especially hydrophobic interaction chromatography (HIC), have also been used. For instance, novel PET capillary‐channeled polymer fibers have been used for HIC with similar results regarding exosome size distribution and number density as those obtained by centrifugation 114. However, reports on other chromatographic modes being employed for this particular application are scarce. In this regard, it should be mentioned that most reports using chromatography as a tool to separate exosomes use different prepacked gravity‐based commercial columns (i.e., in most reviewed cases the columns are not connected to chromatographic equipment). This suggests possible vesicle damage due to pressure changes that chromatographic equipment may exert over them. The buffers needed to operate other chromatographic modes besides SEC, like HIC or ion‐exchange chromatography, may also have implications on the viability of the exosomes once they are processed. Therefore, although chromatography represents an interesting strategy to obtain pure exosome samples from different biological sources, more research is needed, especially if high‐throughput strategies are needed to acquire sufficient amounts of these vesicles for all the possible applications that are arising.

### 2.3 Precipitation

Exosome precipitation is a recently developed technique that is now the second most used isolation method after ultracentrifugation, most likely because it is fairly easy to perform and does not require specialized or expensive equipment 68. This technique was originally developed to isolate viruses and other macromolecules, but exosomes can also be settled from biological fluids using the same principles 115. Most precipitation methods consist on mixing the sample, which can be either a biological fluid or cell culture medium, with a hydrophilic polymer. After mixing, the sample is incubated overnight at 4ºC and afterwards low speed centrifugation is used to precipitate the exosomes which are later resuspended in the preferred buffer for further analysis 116. Protamine, sodium acetate, and organic solvents can also be used for precipitation procedures 117, 118.

Nowadays, exosome precipitation can be achieved with several commercially available reagents like ExoQuick® (System Biosciences), Total Exosome Isolation Reagent® (Invitrogen) and Exosome Purification Kit® (Norgen Biotek), among others. Most of the kits are also available for urine, plasma, serum, cerebrospinal fluid, and cell culture medium matrixes. Precipitation by these reagents is achieved by forcing the exosomes out of solution and trapping them in a porous mesh that facilitates their pelleting at low speed centrifugation. Most available kits contain polyethylene glycol as the hydrophilic compound that excludes water molecules and forms the network in which the exosomes sediment 111, 119.

For instance, this technique was used to isolate exosomes from serum samples to study non‐coding RNAs as biomarkers for human hepatocellular carcinoma. Serum was centrifuged at 3000 rpm for 10 min and then the ExoQuick® isolation solution was added to the supernatant in an appropriate volume. The sample was incubated overnight and centrifuged at 1500 × *g* for 30 min. The pellet was then resuspended in 100–500 µL of PBS. The procedure successfully yielded vesicles for further RNA isolation 120. It was also shown that precipitation with ExoQuick® was a highly reproducible and efficient method. When compared to ultracentrifugation, precipitation resulted in a higher number of particles 121. Ultracentrifugation showed higher levels of protein amount than precipitation. However, ultracentrifuged samples were contaminated with albumin and IgG. Both methods showed equivalent expression of CD9, IAMP2, and Grp94.

In another example, exosomes were isolated from serum using the Total Exosome Isolation Reagent® kit for the study of prostate cancer 122. The reagent was added to the serum in an appropriate volume, incubated for 1 h and centrifuged at 10 000 × *g* for 10 min at room temperature. The pellet was re‐suspended in PBS. Exosomes were identified using transmission electron microscopy and quantified by nanoparticle tracking analysis. CD81 and CD63 were identified by Western Blot and flow cytometry analysis. RNA was extracted and quantified by qRT‐PCR showing a differential expression profile before and after radiation treatment, suggesting a promising new biomarker that needs to be further studied.

It has also been shown that precipitation‐based methods resulted in the highest exosome, miRNA, and mRNA yields from urinary samples when compared to ultracentrifugation and filtration, making precipitation ideal for RNA and proteomic analysis from this matrix 123. Exosomes, precipitated from urine, were quantified using a CD9 ELISA, while miRNAs and mRNAs were assessed by qPCR.

There appears to be a consensus that precipitation‐based methods yield the highest number of extracellular vesicles but with low purity, as it was found when compared to column‐based methods 116. Thus, another approach consists on the incorporation of these methods after precipitation to further purify the sample.

Precipitation is easy to perform, fast, and it does not require an ultracentrifuge or any other expensive equipment. Moreover, several studies support the fact that this method yields the highest number of exosomes, total protein, and RNA 116, 123, 124. Thus, it is considered a cost‐effective technique.

It is important to mention that it has been shown that exosomes isolated by precipitation preserve their biological function, as demonstrated by in vivo particle tracking analysis and miRNA transference assessment 66, 125. Moreover, in these studies, precipitation outperformed ultracentrifugation regarding the number of particles, while showing the same size distribution when compared to ultrafiltration and ultracentrifugation. Furthermore, since precipitation yields the highest total protein and RNA amount, it is also considered the ideal method for RNA analysis. Electron microscopy has demonstrated the preservation of exosome size and morphology after isolation by this technique. Also, precipitation‐based exosome isolation kits are compatible with physiological pH range.

This method is very promising in the clinical context since it does not require sophisticated equipment and processing times are much shorter when compared to other methods, making it ideal for bedside or point‐of‐care diagnostics. Nonetheless, low purity is a key disadvantage. Coisolation of non‐vesicular contaminants such as lipoproteins and ribonucleoprotein complexes, albumin, immunoglobulins and other soluble proteins is unavoidable 126. Contamination with other vesicles is also expected. Unfortunately, this contamination may interfere with further biochemical and immunological analysis. In this context, the need for further purification has produced modified protocols that include pre‐ and post‐isolation cleaning steps, lengthening originally fast procedures 127, and increasing workload and costs as well. Modified precipitation‐based protocols are usually coupled to centrifugation or ultracentrifugation also reducing exosome yields 61.

In addition to coisolation of nonvesicular contaminants and other vesicles, recent studies have found exosome aggregation after precipitation 128, 129. Exosome aggregates may also interfere with downstream analysis. Such findings have led to recommend precipitation for RNA analysis but the alternative use of other methods, such as ultracentrifugation, for protein and biochemical assessment.

New precipitation‐based approaches consist of coating magnetic Fe_3_O_4_ nanoparticles with polyethylene glycol 130. Such strategy has resulted in the removal of albumin and various immunoglobulins, while preserving the number of particles, particle size distribution and CD63 and CD9 expression in the sample. Novel strategies, that address the main disadvantages of precipitation, are required to improve the capabilities of this technique. This is crucial to fulfil the potential of precipitation as a method with serious clinical implications.

### 2.4 Immunoaffinity procedures

So far, we have mentioned immunoblotting as a mean to characterize or to identify exosomes after isolation. However, immunoaffinity techniques can also be used to selectively isolate exosomes from complex biological fluids. Immunoaffinity approaches take advantage of the many proteins, receptors, lipids, and polysaccharides that are present in the outer surface of exosomes. This technique exploits the highly specific affinity interactions that occur between an antigen and an antibody. All the molecules in the surface of exosomes are potential ligands. Nonetheless, exosome biomarkers ideally are highly concentrated or only present on the exosome membrane and lack free counterparts. Proposed exosome biomarkers include tetraspanins, heat shock proteins and MHC antigens, for example, CD9, CD10, CD24, CD63, CD81, EpCAM, Alix, AQP2, FLT1, TSG101, and HSP70, to name a few 131, 132. Also, heparin has been proposed to bind exosomes 133. Even lectins have been used to selectively identify and isolate specific subpopulations of these types of vesicles 134.

Immunoaffinity techniques are usually coupled to other strategies that integrate the specific selection of exosomes with a physical separation or isolation procedure 135, 136. Such strategies allow the enrichment of the sample with exosomes or the depletion of unwanted vesicles. For instance, it was shown that tumor‐derived exosomes can be captured using mAb 763.74 specific for the CDPG4 epitope unique of melanoma cells 137. After monoclonal antibody production, characterization, and biotinylation, the mAb 763.74 was incubated with previously extracted exosomes for 12 h at 4°C. Tumor‐derived exosomes were captured using streptavidin‐coated magnetic beads. The bead‐exosome complexes were then collected using a magnet. Such complex was further used for the fluorescent detection of the antibody with flow cytometry. The data showed that the capture of tumor‐derived exosomes with the anti‐CSPG4 mAb was highly reproducible, with an intra‐class correlation coefficient of 0.98 with the 95% prediction interval at ±5.8.

Commercially available exosome isolation kits have been compared to immunoaffinity purification for the isolation of prostate derived vesicles from plasma, using atomic force microscopy and nanoscale flow cytometry. Immunoaffinity approaches resulted in the elimination of a significant portion of plasma proteins from the sample and a higher yield of prostate‐derived exosomes 138.

Novel methods include modifications of the exosome membrane. A method combining immunoaffinity and lipid membrane surface modification was developed for the isolation and quantification of exosomes. Exosomes are captured, as previously mentioned, with immunomagnetic beads, and then a B‐Chol‐labeled DNA was anchored to the exosome membrane. The sticky end of the anchor initiated the HRP‐linked hybridization chain reaction for signal amplification, easily measured with UV‐Vis spectrometry. This method detected 2.2 × 10^3^ exosomes per microliter, with 100% higher sensitivity compared to ELISA 139. It is believed that assembled assays, which can exploit several proteins and antibodies for plate and bead functionalization, may allow faster quantification and validation of exosomes over current techniques.

Several studies have also been performed to compare immunoaffinity procedures with other isolation methodologies. For instance, a study to compare ultracentrifugation, density‐gradient isolation, and immunoaffinity capture methods was performed. Using the colorectal cancer cell line LIM1863, the methods were evaluated by electron microscopy and immunoblotting for Alix, TSG101 and HSP70. Although, all protocols contained 40–150 nm vesicles that were positive for exosome markers, the proteomic analysis based on the MS/MS spectra identified the immunoaffinity capture methods as the most effective 60. This method has also been shown to yield good quality exosomes for further analysis, as it was shown after isolation of exosomes from serum using magnetic beads. Exosomal miRNAs were extracted after mixing the beads with a solution of a nonionic detergent and salt, and heating. qRT‐PCR was then used to analyze the sample. In another example, a comparison between ultracentrifugation, polymer‐based precipitation and this technique was performed. Findings showed that immunomagnetic capture isolated eight times more exosomes than ultracentrifugation and two times more than polymer‐based precipitation. Moreover, cellular uptake experiments showed that captured exosomes retained biological activity 140.

The main advantage of the immunoaffinity procedures used with this purpose is the better quality and purity of the resulting isolated exosomes 141. Since antigen‐antibody interactions are highly specific, this technique is very useful to selectively isolate a subpopulation of extracellular vesicles without contaminants. Also, this technique is compatible with routine lab‐bench equipment. Nonetheless, preparation of antibodies and magnetic beads among other immunoaffinity strategies requires expertise, not to mention expensive materials and reagents. It should also be noted that immunoaffinity methods are almost invariably hyphenated to other techniques 126. Initially, exosomal content and RNA yields appeared to be the main issue with this strategy. However, the latest reports show a comparable or even greater yield when compared to ultracentrifugation and precipitation‐based methods 140. Besides higher capture efficiency and sensitivity, this approach can also be easily scaled due to the lack of limitations regarding the initial sample volume.

### 2.5 Microfluidics

The advent of microfabrication technology has increased the interest in microscale processes capable of performing biological analysis and purification procedures with high accuracy and specificity. Microfluidic devices, based on the manipulation of fluids at the micrometer scale, are an innovative strategy to efficiently isolate exosomes minimizing time, equipment and costs 142. Nowadays, lab‐on‐a‐chip devices that involve the integration of functional components explore the behavior of exosomes at the microscale. Exosome manipulation techniques based on microdevices include approaches like electrophoresis, dielectrophoresis, acoustics, magnetism, and immunoaffinity 97, 143, 144. Furthermore, devices that combine two or more separation techniques have been designed.

For instance, a two‐stage magnetic‐based microdevice, which integrates isolation and *in situ* electrochemical analysis of exosomes from blood samples has been developed 145. The device integrates an array of Y‐shaped posts, to enhance Tim4‐modified magnetic beads‐exosome interaction and a cascading ITO electrode. The capture method involves magnetic enrichment on the surface of the electrode. In this device, the electrode integrated a signal transduction strategy based on a sensor containing CD63 and a mimicking DNAzyme sequence. CD63‐positive exosomes enhance the production of H_2_O_2_ by NADH oxidation accompanied by signal enhancement. This device captured tumor‐derived exosomes within 3.5 h in 30 µL samples and results were confirmed by ELISA and Western Blot 145.

Size‐exclusion methods can also be integrated in microdevices. Nanoporous membranes or nanoarrays can be built inside the channel. In this case, exosomes are isolated by a pressure‐driven filtration process. One of the main advantages of this type of devices is their capability to remove over 95% of the contaminants. Results are good enough to be confirmed by Western Blot and RT‐PCR 144.

Some authors classify the exosome isolation mechanism within the microdevice as passive (static sorting) and active (dynamic sorting) 68, 142. Passive sorting techniques include immunoaffinity, size‐exclusion, and flow‐induced methods. On the other hand, active sorting includes electroactive separation, immunomagnetic isolation, and acoustofluidics. Both active and passive sorting may also be combined in a single device.

Electroactive strategies are somewhat simpler than other techniques since no antibody affinity or other biochemical methods are needed. Moreover, no pumps or moving parts are required since the separation occurs based on charge‐to‐mass ratios. Shi et al. used a dielectrophoretic approach to design a low voltage nanopipette for entrapment of exosomes from plasma 146. This study showed exosome entrapment at a glass pipette tip using direct current at a minimum 10 V/cm. Maximum enrichment was achieved after 1 h of applied voltage, but particles were detected after 100 seconds. Results were confirmed by detected fluorescence intensity measurements 146. Such results opened the door to the further development of dielectrophoretic entrapment of exosomes and other electroactive strategies.

As known, microfluidic devices have the potential to replace expensive and spacious laboratory equipment. Also, in contrast to other methods the required volume of the sample and reagents is considerably lower, and the process may be automated and performed in a very short period of time 147. These isolation techniques also have the potential of high throughput and customization 148. However, it should be noted that the device design is key for scalability and will impact process standardization. Nonetheless, no microfluidic device is free of shortcomings. For instance, exosome yield in immunoaffinity‐based devices or microporous systems is lower when compared to ultracentrifugation or precipitation 149. Moreover, although trained personal is not required to operate microdevices, reproducibility remains a challenge 150. Analysis of the sample after isolation by microfluidics also needs to be standardized since results are usually not consistent 58, 146, 148, 149, 151.

The physical properties of exosomes are of interest when discussing the disadvantages of microdevices. Exosomes tend to aggregate, so device blocking is common 152, 153. Furthermore, the effect of microfluidic forces on exosome integrity needs to be studied. Also, functionalization of such cellular nanoparticles within the device may challenge post‐isolation analysis 151.

Finally, the movement from basic research to the clinical or application setting is a difficult quest. Although the potential for clinical or other types of applications is limitless, micro‐devices need to be more robust and effective than the available standard methods. The full potential of this technology will be seen when the collaboration between engineers and clinicians or other experts results in overcoming the challenges posed by different application settings.

## 3 The future of exosome isolation procedures

Exosome isolation remains a challenge for biomedical research. There is still no consensus over which purification technique produces the best results and there is intense competition within the field. Moreover, an accurate comparison between methods cannot be easily made because of the inherent exosome complexity 12, 154. However, exosome isolation has evolved over the last decade. Current methods have made progress in elucidating the role of exosomes in health and disease 67, 155; and several characterization techniques have been employed to successfully prove the presence of exosomes in enriched samples from a variety of complex biological fluids 16, 156. Yet, the gap between basic research and clinical applications remains wide. Current methods are not scalable for the clinical setting. Moreover, reasonable throughput and validation are required for bedside technology. In addition to avoid the use of specialized equipment, an ideal isolation method should be fast, reproducible, easy to perform, and flexible (i.e., must work with several biological matrices). Moreover, coisolation of contaminants should be minimum since contamination is the most common complication of current isolation techniques 58. Almost invariably, coisolation of other vesicles and non‐vesicular molecules occurs, interfering with data comparison between research laboratories 123.

Although, ultracentrifugation is currently the gold standard for exosome isolation. There is no ideal method that fits all purposes. The selection of the procedure usually depends on the capabilities and resources of each research team and sacrifices must be made in terms of recovery, purity or work load. Moreover, downstream analysis may be compromised by the isolation technique that is chosen 90. In this sense, the final selection of the most suitable technique for exosome isolation and purification needs to consider the effects that the methodology may exert over the sample integrity particularly for the intended final use. For instance, recovery techniques such as ultracentrifugation and filtration tend to render a population of “saucer‐like” or “deflated‐football” shaped vesicles that might no longer be useful 157. Furthermore, the integrity of the exosomal cargo to unravel exosome‐specific functions and biomarkers should also be considered even when no apparent degradation is present 158. This is especially true for microfluidic techniques or after isolation when exosomes are stored under freezing or other harsh conditions 159.

In this work, we have discussed the advantages and disadvantages of the most commonly used exosome extraction methods and some comparisons have been made. In general terms, the exosome isolation method selection should depend on the downstream analysis requirements, but it should be noted that technique combinations can improve isolation efficiency 72, 96, 107. Nonetheless, additional time, reagents, and cost must be considered. Furthermore, additional separation procedures raise the error rate and may reduce exosome recovery yields. Thus, a proposed solution consists of integrating isolation and analysis procedures. In this sense, since immunoaffinity techniques have proven that isolation and characterization can be done in a few easy steps 126 it is believed that these research lines will be considerably important in the following years. A one‐size‐fits‐all exosome recovery and purification method is still something difficult to develop, but the standardization of existing protocols that combine techniques for the resolution of a particular problem is the key. It is clear that the future of exosome isolation is a translational technology and that the development of robust and high‐throughput protocols is required for the upcoming applications in all types of settings.

## 4 Concluding remarks

There are currently different strategies being used in the isolation and purification of exosomes whose selection depends on the intended application for the exosomal extract. To date, it is difficult to identify a strategy that yields the highest quality and properties of the isolated exosomes and usually a combination of the different methodologies is required to achieve the best results. Ultracentrifugation, to our belief, will continue to be one of the most used methodologies in exosome procurement. However, novel emerging strategies involving immunoaffinity and microdevices are appearing with interesting results and as a viable option to obtain these important nanoparticles.

As it has been mentioned, exosome‐related applications are gaining attention by the scientific community since these vesicles can be used as carriers for different formulations with low side‐effects and high specificity. In this line, one of the most promising applications refers to the use of these nanovesicles as carriers for gene therapies in tissue restoration and cancer applications. However, in order for these approaches to find a potential market and profitable application, exosome isolation and purification methods need to be further studied and scaled. This work presented some of the most used techniques for this purpose to date. Nonetheless, it is believed that in the following years new advances will be incorporated in these procedures with improvements to the current strategies or with completely new approaches. Furthermore, the development of exosome purification technologies will be closely related to the design of novel applications for these interesting and versatile nanoparticles.

*The authors have declared no conflict of interest*.

## References

[1] Bloemendal, S. , Kück, U. , Naturwissenschaften 2013, 100, 3–19. 23128987 10.1007/s00114-012-0988-z

[2] Van Niel, G. , Porto‐Carreiro, I. , Simoes, S. , Raposo, G. , J. Biochem. 2006, 140, 13–21. 16877764 10.1093/jb/mvj128

[3] Keller, S. , Sanderson, M. P. , Stoeck, A. , Altevogt, P. , Immunol. Lett. 2006, 107, 102–108. 17067686 10.1016/j.imlet.2006.09.005

[4] Shah, R. , Patel, T. , Freedman, J. , N. Engl. J. Med. 2018, 379, 958–966. 30184457 10.1056/NEJMra1704286

[5] Webber, J. , Yeung, V. , Clayton, A. , Semin. Cell Dev. Biol. 2015, 40, 27–34. 25662446 10.1016/j.semcdb.2015.01.013

[6] Sahoo, S. , Losordo, D. W. , Circ. Res. 2014, 114, 333–344. 24436429 10.1161/CIRCRESAHA.114.300639

[7] Melo, S. A. , Luecke, L. B. , Kahlert, C. , Fernandez, A. F. , Gammon, S. T. , Kaye, J. , LeBleu, V. S. , Mittendorf, E. A. , Weitz, J. , Rahbari, N. , Reissfelder, C. , Pilarsky, C. , Fraga, M. F. , Piwnica‐Worms, D. , Kalluri, R. , Nature 2015, 523, 177–182. 26106858 10.1038/nature14581 PMC4825698

[8] Barile, L. , Vassalli, G. , Pharmacol. Ther. 2017, 174, 63–78. 28202367 10.1016/j.pharmthera.2017.02.020

[9] Soung, Y. H. , Ford, S. , Zhang, V. , Chung, J. , Cancer 2017, 9, 8. 10.3390/cancers9010008 PMC5295779 28085080

[10] Thakur, B. K. , Zhang, H. , Becker, A. , Matei, I. , Huang, Y. , Costa‐Silva, B. , Zheng, Y. , Hoshino, A. , Brazier, H. , Xiang, J. , Williams, C. , Rodriguez‐Barrueco, R. , Silva, J. M. , Zhang, W. , Hearn, S. , Elemento, O. , Paknejad, N. , Manova‐Todorova, K. , Welte, K. , Bromberg, J. , Peinado, H. , Lyden, D. , Cell Res. 2014, 24, 766–769. 24710597 10.1038/cr.2014.44 PMC4042169

[11] Johnsen, K. B. , Gudbergsson, J. M. , Skov, M. N. , Pilgaard, L. , Moos, T. , Duroux, M. , Biochim. Biophys. Acta 2014, 1846, 75–87. 24747178 10.1016/j.bbcan.2014.04.005

[12] Chaput, N. , Théry, C. , Semin. Immunopathol. 2011, 33, 419–440. 21174094 10.1007/s00281-010-0233-9

[13] Greening, D. W. , Gopal, S. K. , Xu, R. , Simpson, R. J. , Chen, W. , Semin. Cell Dev. Biol. 2015, 40, 72–81. 25724562 10.1016/j.semcdb.2015.02.009

[14] Cheng, L. , Wang, Y. , Huang, L. , Mol. Ther. 2017, 25, 1665–1675. 28284981 10.1016/j.ymthe.2017.02.007 PMC5498801

[15] Mathivanan, S. , Ji, H. , Simpson, R. J. , J. Proteomics 2010, 73, 1907–1920. 20601276 10.1016/j.jprot.2010.06.006

[16] Petersen, K. E. , Manangon, E. , Hood, J. L. , Wickline, S. A. , Fernandez, D. P. , Johnson, W. P. , Gale, B. K. , Anal. Bioanal. Chem. 2014, 406, 7855–7866. 25084738 10.1007/s00216-014-8040-0 PMC4492512

[17] Frühbeis, C. , Fröhlich, D. , Kuo, W. P. , Amphornrat, J. , Thilemann, S. , Saab, A. S. , Kirchhoff, F. , Möbius, W. , Goebbels, S. , Nave, K. A. , Schneider, A. , Simons, M. , Klugmann, M. , Trotter, J. , Krämer‐Albers, E. M. , PLoS Biol. 2013, 11, e1001604. 23874151 10.1371/journal.pbio.1001604 PMC3706306

[18] Hu, M. , Hong, L. , Liu, C. , Hong, S. , He, S. , Zhou, M. , Huang, G. , Chen, Q. , Sci. Rep. 2019, 9, 4206. 30862846 10.1038/s41598-019-41007-5 PMC6414536

[19] Xin, H. , Wang, F. , Li, Y. , Lu, Q.‐E. , Cheung, W. L. , Zhang, Y. , Zhang, Z. G. , Chopp, M. , Cell Transplant. 2017, 26, 243–257. 27677799 10.3727/096368916X693031 PMC5303172

[20] Wang, Y. , Balaji, V. , Kaniyappan, S. , Krüger, L. , Irsen, S. , Tepper, K. , Chandupatla, R. , Maetzler, W. , Schneider, A. , Mandelkow, E. , Mandelkow, E.‐M. , Mol. Neurodegener. 2017, 12, 5. 28086931 10.1186/s13024-016-0143-y PMC5237256

[21] Lee, S. , Mankhong, S. , Kang, J.‐H. , Int. J. Mol. Sci. 2019, 20, 1728. 10.3390/ijms20071728 PMC6479979 30965555

[22] Lyu, L. , Wang, H. , Li, B. , Qin, Q. , Qi, L. , Nagarkatti, M. , Nagarkatti, P. , Janicki, J. S. , Wang, X. L. , Cui, T. , J. Mol. Cell Cardiol. 2015, 89, 268–279. 26497614 10.1016/j.yjmcc.2015.10.022 PMC4988239

[23] Bang, C. , Batkai, S. , Dangwal, S. , Gupta, S. K. , Foinquinos, A. , Holzmann, A. , Just, A. , Remke, J. , Zimmer, K. , Zeug, A. , Ponimaskin, E. , Schmiedl, A. , Yin, X. , Mayr, M. , Halder, R. , Fischer, A. , Engelhardt, S. , Wei, Y. , Schober, A. , Fiedler, J. , Thum, T. , J. Clin. Invest. 2014, 124, 2136–2146. 24743145 10.1172/JCI70577 PMC4001534

[24] Bellin, G. , Gardin, C. , Ferroni, L. , Chachques, J. C. , Rogante, M. , Mitrečić, D. , Ferrari, R. , Zavan, B. , Cells 2019, 8, 166. 10.3390/cells8020166 PMC6406975 30781555

[25] Chen, L. , Chen, R. , Kemper, S. , Brigstock, D. R. , J. Cell Commun. Signal. 2018, 12, 343–357. 29063370 10.1007/s12079-017-0421-7 PMC5842184

[26] Sato, K. , Meng, F. , Glaser, S. , Alpini, G. , J. Hepatol. 2016, 65, 213–221. 26988731 10.1016/j.jhep.2016.03.004 PMC4912847

[27] Eguchi, A. , Feldstein, A. E. , Liver Res. 2018, 2, 30–34. 30345152 10.1016/j.livres.2018.01.001 PMC6190912

[28] Masyuk, A. I. , Masyuk, T. V. , Larusso, N. F. , J. Hepatol. 2013, 59, 621–625. 23557871 10.1016/j.jhep.2013.03.028 PMC3831338

[29] Nojima, H. , Freeman, C. M. , Schuster, R. M. , Japtok, L. , Kleuser, B. , Edwards, M. J. , Gulbins, E. , Lentsch, A. B. , J. Hepatol. 2016, 64, 60–68. 26254847 10.1016/j.jhep.2015.07.030 PMC4843792

[30] Vizoso, F. J. , Eiro, N. , Cid, S. , Schneider, J. , Perez‐Fernandez, R. , Int. J. Mol. Sci. 2017, 18, 1852. 10.3390/ijms18091852 PMC5618501 28841158

[31] Chang, Y. H. , Wu, K. C. , Harn, H. J. , Lin, S. Z. , Ding, D. C. , Cell Transplant. 2018, 27, 349–363. 29692195 10.1177/0963689717723636 PMC6038041

[32] Lai, R. C. , Yeo, R. W. Y. , Lim, S. K. , Semin. Cell Dev. Biol. 2015, 40, 82–88. 25765629 10.1016/j.semcdb.2015.03.001

[33] Fatima, F. , Nawaz, M. , Chin. J. Cancer. 2015, 34, 541–553. 26369565 10.1186/s40880-015-0051-5 PMC4593342

[34] Forterre, A. , Jalabert, A. , Berger, E. , Baudet, M. , Chikh, K. , Errazuriz, E. , De Larichaudy, J. , Chanon, S. , Weiss‐Gayet, M. , Hesse, A. M. , Record, M. , Geloen, A. , Lefai, E. , Vidal, H. , Couté, Y. , Rome, S. , PLoS One 2014, 9, e84153. 24392111 10.1371/journal.pone.0084153 PMC3879278

[35] Forterre, A. , Jalabert, A. , Chikh, K. , Pesenti, S. , Euthine, V. , Granjon, A. , Errazuriz, E. , Lefai, E. , Vidal, H. , Rome, S. , Cell Cycle 2014, 13, 78‐89. 24196440 10.4161/cc.26808 PMC3925739

[36] Friedman, A. , Hao, W. , Bull. Math. Biol. 2018, 80, 1111–1133. 28382422 10.1007/s11538-017-0254-9

[37] Takikawa, T. , Masamune, A. , Yoshida, N. , Hamada, S. , Kogure, T. , Shimosegawa, T. , Pancreas 2017, 46, 19–27. 27841793 10.1097/MPA.0000000000000722

[38] Cianciaruso, C. , Phelps, E. A. , Pasquier, M. , Hamelin, R. , Demurtas, D. , Ahmed, M. A. , Piemonti, L. , Hirosue, S. , Swartz, M. A. , De Palma, M. , Hubbell, J. A. , Baekkeskov, S. , Diabetes 2017, 66, 460–473. 27872147 10.2337/db16-0671

[39] Delić, D. , Eisele, C. , Schmid, R. , Baum, P. , Wiech, F. , Gerl, M. , Zimdahl, H. , Pullen, S. S. , Urquhart, R. , PLoS One 2016, 11, e0150154. 26930277 10.1371/journal.pone.0150154 PMC4773074

[40] Sole, C. , Cortes‐Hernandez, J. , Felip, M. L. , Vidal, M. , Ordi‐Ros, J. , Nephrol. Dial. Transplant. 2015, 30, 1488–1496. 26040904 10.1093/ndt/gfv128

[41] Fraser, K. B. , Rawlins, A. B. , Clark, R. G. , Alcalay, R. N. , Standaert, D. G. , Liu, N. , West, A. B. , Mov. Disord. 2016, 31, 1543–1550. 27297049 10.1002/mds.26686 PMC5053851

[42] Feng, Y. , Lv, L. L. , Wu, W. J. , Li, Z. L. , Chen, J. , Ni, H. F. , Zhou, L. T. , Tang, T. T. , Wang, F. M. , Wang, B. , Chen, P. S. , Crowley, S. D. , Liu, B. C. , Am. J. Pathol. 2018, 188, 2542–2552. 30142333 10.1016/j.ajpath.2018.07.017

[43] Øverbye, A. , Skotland, T. , Koehler, C. J. , Thiede, B. , Seierstad, T. , Berge, V. , Sandvig, K. , Llorente, A. , Oncotarget 2015, 6, 30357–30376. 26196085 10.18632/oncotarget.4851 PMC4745805

[44] Tan, L. , Wu, H. , Liu, Y. , Zhao, M. , Li, D. , Lu, Q. , Autoimmunity 2016, 49, 357–365. 27259064 10.1080/08916934.2016.1191477

[45] Buschow, S. I. , van Balkom, B. W. M. , Aalberts, M. , Heck, A. J. R. , Wauben, M. , Stoorvogel, W. , Immunol. Cell Biol. 2010, 88, 851–856. 20458337 10.1038/icb.2010.64

[46] Théry, C. , Duban, L. , Segura, E. , Véron, P. , Lantz, O. , Amigorena, S. , Nat. Immunol. 2002, 3, 1156–1162. 12426563 10.1038/ni854

[47] Bhatnagar, S. , Schorey, J. S. , J. Biol. Chem. 2007, 282, 25779–25789. 17591775 10.1074/jbc.M702277200 PMC3636815

[48] Clayton, A. , Turkes, A. , Dewitt, S. , Steadman, R. , Mason, M. D. , Hallett, M. B. , FASEB J. 2004, 18, 977–979. 15059973 10.1096/fj.03-1094fje

[49] Hock, A. , Miyake, H. , Li, B. , Lee, C. , Ermini, L. , Koike, Y. , Chen, Y. , Määttänen, P. , Zani, A. , Pierro, A. , J. Pediatr. Surg. 2017, 52, 755–759. 28188035 10.1016/j.jpedsurg.2017.01.032

[50] Admyre, C. , Johansson, S. M. , Qazi, K. R. , Filen, J.‐J. , Lahesmaa, R. , Norman, M. , Neve, E. P. A. , Scheynius, A. , Gabrielsson, S. , J. Immunol. 2014, 179, 1969–1978. 10.4049/jimmunol.179.3.1969 17641064

[51] Qin, W. , Tsukasaki, Y. , Dasgupta, S. , Mukhopadhyay, N. , Ikebe, M. , Sauter, E. R. , Clin. Cancer Res. 2016, 22, 4517–4524. 27060153 10.1158/1078-0432.CCR-16-0135

[52] Näslund, T. I. , Paquin‐Proulx, D. , Paredes, P. T. , Vallhov, H. , Sandberg, J. K. , Gabrielsson, S. , Aids 2014, 28, 171–180. 24413309 10.1097/QAD.0000000000000159

[53] Yu, S. , Cao, H. , Shen, B. , Feng, J. , Oncotarget 2015, 6, 37151–37168. 26452221 10.18632/oncotarget.6022 PMC4741921

[54] Zhang, X. , Yuan, X. , Shi, H. , Wu, L. , Qian, H. , Xu, W. , J. Hematol. Oncol. 2015, 8, 1–14. 26156517 10.1186/s13045-015-0181-x PMC4496882

[55] Huang‐Doran, I. , Zhang, C. Y. , Vidal‐Puig, A. , Trends Endocrinol. Metab. 2017, 28, 3–18. 27810172 10.1016/j.tem.2016.10.003

[56] Record, M. , Subra, C. , Silvente‐poirot, S. , Poirot, M. , Biochem. Pharmacol. 2011, 81, 1171–1182. 21371441 10.1016/j.bcp.2011.02.011

[57] Jella, K. , Nasti, T. , Li, Z. , Malla, S. , Buchwald, Z. , Khan, M. , Vaccines 2018, 6, 69. 10.3390/vaccines6040069 PMC6313856 30261592

[58] Li, P. , Kaslan, M. , Lee, S. H. , Yao, J. , Gao, Z. , Theranostics 2017, 7, 789–804. 28255367 10.7150/thno.18133 PMC5327650

[59] Livshits, M. A. , Khomyakova, E. , Evtushenko, E. G. , Lazarev, V. N. , Kulemin, N. A. , Semina, S. E. , Generozov, E. V. , Govorun, V. M. , Sci. Rep. 2015, 5, 17319. 26616523 10.1038/srep17319 PMC4663484

[60] Tauro, B. J. , Greening, D. W. , Mathias, R. A. , Ji, H. , Mathivanan, S. , Scott, A. M. , Simpson, R. J. , Methods 2012, 56, 293–304. 22285593 10.1016/j.ymeth.2012.01.002

[61] Lobb, R. J. , Becker, M. , Wen, S. W. , Wong, C. S. F. , Wiegmans, A. P. , Leimgruber, A. , Möller, A. , J. Extracell. Vesicles 2015, 4, 27031. 26194179 10.3402/jev.v4.27031 PMC4507751

[62] Muller, L. , Hong, C. S. , Stolz, D. B. , Watkins, S. C. , Whiteside, T. L. , J. Immunol. Meth. 2014, 411, 55–65. 10.1016/j.jim.2014.06.007 PMC4260336 24952243

[63] Thery, C. , Amigorena, S. S. , Raposo, G. G. , Clayton, A. , Curr. Protoc. Cell Biol. 2006, https://www.ncbi.nlm.nih.gov/pubmed/18228490# 10.1002/0471143030.cb0322s30 18228490

[64] Pospichalova, V. , Svoboda, J. , Dave, Z. , Kotrbova, A. , Kaiser, K. , Klemova, D. , Ilkovics, L. , Hampl, A. , Crha, I. , Jandakova, E. , Minar, L. , Weinberger, V. , Bryja, V. , J. Extracell. Vesicles 2015, 4, 1–15. 10.3402/jev.v4.25530 PMC4382613 25833224

[65] Gupta, S. , Rawat, S. , Arora, V. , Kottarath, S. K. , Dinda, A. K. , Vaishnav, P. K. , Nayak, B. , Mohanty, S. , Stem Cell Res. Ther. 2018, 9, 1–12. 29973270 10.1186/s13287-018-0923-0 PMC6033286

[66] Helwa, I. , Cai, J. , Drewry, M. D. , Zimmerman, A. , Dinkins, M. B. , Khaled, M. L. , Seremwe, M. , Dismuke, W. M. , Bieberich, E. , Stamer, W. D. , Hamrick, M. W. , Liu, Y. , PLoS One 2017, 12, 1–23. 10.1371/journal.pone.0170628 PMC5256994 28114422

[67] Li, B. , Li, L. , Zhang, Q. , Zhang, H. , Xiu, R. , Microcirculation 2018, e12515. 30431204 10.1111/micc.12515

[68] Konoshenko, M. Y. , Lekchnov, E. A. , Vlassov, A. V. , Laktionov, P. P. , Biomed Res. Int. 2018, 30, 8545347. 10.1155/2018/8545347 PMC5831698 29662902

[69] Zhang, Y. , Li, M. , Hu, C. , Biochem. Biophys. Res. Commun. 2018, 507, 457–464. 30458987 10.1016/j.bbrc.2018.11.061

[70] Cvjetkovic, A. , Lötvall, J. , Lässer, C. , J. Extracell. Vesicles 2014, 3, 23111. 10.3402/jev.v3.23111 PMC3967015 24678386

[71] Leblanc, P. , Arellano‐Anaya, Z. E. , Bernard, E. , Gallay, L. , Provansal, M. , Lehmann, S. , Schaeffer, L. , Raposo, G. , Vilette, D. , Isolation of Exosomes and Microvesicles from Cell Culture Systems to Study Prion Transmission, Springer, New York 2017, pp. 153–176. 10.1007/978-1-4939-6728-5_11 27943213

[72] Onódi, Z. , Pelyhe, C. , Terézia Nagy, C. , Brenner, G. B. , Almási, L. , Kittel, Á. , Manček‐Keber, M. , Ferdinandy, P. , Buzás, E. I. , Giricz, Z. Front. Physiol. 2018, 9, 1479. 30405435 10.3389/fphys.2018.01479 PMC6206048

[73] Wu, C.‐X. , Liu, Z.‐F. , J. Invest. Dermatol. 2018, 138, 89–97. 28899687 10.1016/j.jid.2017.05.040

[74] Lee, J. , McKinney, K. Q. , Pavlopoulos, A. J. , Han, M. H. , Kim, S.‐H. , Kim, H. J. , Hwang, S. , Clin. Chim. Acta 2016, 462, 118–126. 27609124 10.1016/j.cca.2016.09.001

[75] Lee, T. H. , Chennakrishnaiah, S. , Rak, J. , Cell. Mol. Biol. Lett. 2015, 20, 117–129. 26204397 10.1515/cmble-2015-0003

[76] Jeppesen, D. K. , Hvam, M. L. , Primdahl‐Bengtson, B. , Boysen, A. T. , Whitehead, B. , Dyrskjøt, L. , Orntoft, T. F. , Howard, K. A. , Ostenfeld, M. S. , J. Extracell. Vesicles 2014, 3, 25011. 25396408 10.3402/jev.v3.25011 PMC4224706

[77] Qi, J. , Zhou, Y. , Jiao, Z. , Wang, X. , Zhao, Y. , Li, Y. , Chen, H. , Yang, L. , Zhu, H. , Li, Y. , Cell. Physiol. Biochem. 2017, 42, 2242–2254. 28817816 10.1159/000479998

[78] Barrachina, M. N. , Calderón‐Cruz, B. , Fernandez‐Rocca, L. , García, Á. , Proteomics 2019, 19, 1800247. 10.1002/pmic.201800247 30467982

[79] He, L. , Zhu, D. , Wang, J. , Wu, X. , Int. J. Mol. Med. 2019, 43, 83–90. 30365060 10.3892/ijmm.2018.3944 PMC6257847

[80] Sims, N. R. , Anderson, M. F. , Nat. Protoc. 2008, 3, 1228–1239. 18600228 10.1038/nprot.2008.105

[81] Yamamoto, Y. , Itoh, S. , Yamauchi, Y. , Matsushita, K. , Ikeda, S. , Naruse, H. , Hayashi, M. , J. Cell. Biochem. 2015, 116, 2709–2714. 26105044 10.1002/jcb.25270

[82] Manner, A. , Islinger, M. , Methods Mol. Biol. 2017, 1595, 1–11. 28409446 10.1007/978-1-4939-6937-1_1

[83] Looze, C. , Yui, D. , Leung, L. , Ingham, M. , Kaler, M. , Yao, X. , Wu, W. W. , Shen, R. F. , Daniels, M. P. , Levine, S. J. , Biochem. Biophys. Res. Commun. 2009, 378, 433‐438. 19028452 10.1016/j.bbrc.2008.11.050 PMC2633355

[84] Konadu, K. A. , Huang, M. B. , Roth, W. , Armstrong, W. , Powell, M. , Villinger, F. , Bond, V. , J. Vis. Exp. 2016, 107, 53495. 10.3791/53495 PMC4780996 26780239

[85] de Araújo, M. E. G. , Lamberti, G. , Huber, L. A. , Cold Spring Harb. Protoc. 2015, 11, 1013–1016. 10.1101/pdb.prot083444 26527762

[86] Herrmann, E. , Koch, P. , Riedel, C. U. , Young, W. , Egert, M. , Can. J. Microbiol. 2016, 63, 83–87. 27919161 10.1139/cjm-2016-0483

[87] Street, J. M. , Koritzinsky, E. H. , Glispie, D. M. , Yuen, P. S.T . Urine exosome isolation and characterization, in: Gautier J.‐C. (Ed.), Drug Safety Evaluation: Methods and Protocols, Springer, New York 2017, pp. 413–423. 10.1007/978-1-4939-7172-5_23 PMC9374111 28748478

[88] Raj, D. A. A. , Fiume, I. , Capasso, G. , Pocsfalvi, G. , Kidney Int. 2012, 81, 1263–1272. 22418980 10.1038/ki.2012.25

[89] Rajendran, L. , Honsho, M. , Zahn, T. R. , Keller, P. , Geiger, K. D. , Verkade, P. , Simons, K. , Proc. Natl. Acad. Sci. USA 2006, 103, 11172–11177. 16837572 10.1073/pnas.0603838103 PMC1544060

[90] Yamashita, T. , Takahashi, Y. , Nishikawa, M. , Takakura, Y. , Eur. J. Pharm. Biopharm. 2016, 98, 1–8. 26545617 10.1016/j.ejpb.2015.10.017

[91] Heinemann, M. L. , Vykoukal, J . Sequential Filtration: A Gentle Method for the Isolation of Functional Extracellular Vesicles, in: Kuo W.P., Jia S. (Eds.), Extracellular Vesicles: Methods and Protocols, Springer, New York 2017, pp. 33–41. 10.1007/978-1-4939-7253-1_4 28828646

[92] Musante, L. , Tataruch‐Weinert, D. , Kerjaschki, D. , Henry, M. , Meleady, P. , Holthofer, H. , J. Extracell. Vesicles 2016, 6, 1267896. 28326167 10.1080/20013078.2016.1267896 PMC5328348

[93] Bukong, T. N. , Momen‐Heravi, F. , Kodys, K. , Bala, S. , Szabo, G. , PLoS Pathog. 2014, 10, e1004424. 25275643 10.1371/journal.ppat.1004424 PMC4183590

[94] Chen, C. Y. , Hogan, M. C. , Ward, C. J. , Meth. Enzymol. 2013, 524, 225–241. 23498743 10.1016/B978-0-12-397945-2.00013-5 PMC4028690

[95] Escudier, B. , Dorval, T. , Chaput, N. , ré, F. , Caby, M. P. , Novault, S. , Flament, C. , Leboulaire, C. , Borg, C. , Amigorena, S. , Boccaccio, C. , Bonnerot, C. , Dhellin, O. , Movassagh, M. , Piperno, S. , Robert, C. , Serra, V. , Valente, N. , Le Pecq, J. B. , Spatz, A. , Lantz, O. , Tursz, T. , Angevin, E. , Zitvogel, L. , J. Transl. Med. 2005, 3,10. 15740633 10.1186/1479-5876-3-10 PMC554765

[96] Benedikter, B. J. , Bouwman, F. G. , Vajen, T. , Heinzmann, A. C. A. , Grauls, G. , Mariman, E. C. , Wouters, E. F. M. , Savelkoul, P. H. , Lopez‐Iglesias, C. , Koenen, R. R. , Rohde, G. G. U. , Stassen, F. R. M. , Sci. Rep. 2017, 7, 15297. 29127410 10.1038/s41598-017-15717-7 PMC5681555

[97] Huang, T. , Banizs, A. B. , Shi, W. , Klibanov, A. L. , He, J. , J. Circ. Biomarkers 2015, 4, 6. 10.5772/61148 PMC5572986 28936242

[98] Gheinani, A. H. , Vögeli, M. , Baumgartner, U. , Vassella, E. , Draeger, A. , Burkhard, F. C. , Monastyrskaya, K. , Sci. Rep. 2018, 8, 3945. 29500443 10.1038/s41598-018-22142-x PMC5834546

[99] Eliaz, D. , Kannan, S. , Shaked, H. , Arvatz, G. , Tkacz, I. D. , Binder, L. , Waldman Ben‐Asher, H. , Okalang, U. , Chikne, V. , Cohen‐Chalamish, S. , Michaeli, S. , PLoS Pathog. 2017, 13, e1006245. 28257521 10.1371/journal.ppat.1006245 PMC5352147

[100] Taylor, D. D. , Shah, S. , Methods 2015, 87, 3–10. 25766927 10.1016/j.ymeth.2015.02.019

[101] Bryzgunova, O. E. , Zaripov, M. M. , Skvortsova, T. E. , Lekchnov, E. A. , Grigor'eva, A. E. , Zaporozhchenko, I. A. , Morozkin, E. S. , Ryabchikova, E. I. , Yurchenko, Y. B. , Voitsitskiy, V. E. , Laktionov, P. P. , PLoS One 2016, 11, e0157566. 27305142 10.1371/journal.pone.0157566 PMC4909321

[102] Channavajjhalaa, S. K. , Rossatoa, M. , Morandini, F. , Castagna, A. , Pizzolo, F. , Bazzoni, F. , Olivieri, O. , Clin. Chem. Lab. Med. 2014, 3, 345–354. 10.1515/cclm-2013-0562 24101370

[103] Cheruvanky, A. , Zhou, H. , Pisitkun, T. , Kopp, J. B. , Knepper, M. A. , Yuen, P. S. T. , Star, R. A. , Am. J. Physiol. Renal Physiol. 2007, 292, F1657–F1661. 17229675 10.1152/ajprenal.00434.2006 PMC2271070

[104] Batrakova, E. V. , Kim, M. S. , J. Controlled Release 2015, 219, 396–405. 10.1016/j.jconrel.2015.07.030 PMC4656109 26241750

[105] Musante, L. , Tataruch, D. , Gu, D. , Benito‐Martin, A. , Calzaferri, G. , Aherne, S. , Holthofer, H. , Sci. Rep. 2014, 4, 7532. 25532487 10.1038/srep07532 PMC4274508

[106] Zeringer, E. , Barta, T. , Li, M. , Vlassov, A. V. , Cold Spring Harb. Protoc. 2015, 319–323. 25834266 10.1101/pdb.top074476

[107] Corso, G. , Mäger, I. , Lee, Y. , Görgens, A. , Bultema, J. , Giebel, B. , Wood, M. J. A. , Nordin, J. Z. , Andaloussi, S. E. L. , Sci. Rep. 2017, 7, 11561. 28912498 10.1038/s41598-017-10646-x PMC5599601

[108] Feng, Y. , Huang, W. , Wani, M. , Yu, X. , Ashraf, M. , PLoS One 2014, 9, e88685. 24558412 10.1371/journal.pone.0088685 PMC3928277

[109] Rood, I. M. , Deegens, J. K. J. , Merchant, M. L. , Tamboer, W. P. M. , Wilkey, D. W. , Wetzels, J. F. M. , Klein, J. B. , Kidney Int. 2010, 78, 810–816. 20686450 10.1038/ki.2010.262

[110] Koh Y. Q. , Almughlliq F. B. , Vaswani K. , Peiris H. N. , Mitchell, D. M. , Front. Biosci. 2018, 23, 865–874. 10.2741/4621 28930577

[111] Gámez‐Valero, A. , Monguió‐Tortajada, M. , Carreras‐Planella, L. , Franquesa, M. , Beyer, K. , Borràs, F. E. , Sci. Rep. 2016, 6, 33641. 27640641 10.1038/srep33641 PMC5027519

[112] Nordin, J. Z. , Lee, Y. , Vader, P. , Mäger, I. , Johansson, H. J. , Heusermann, W. , Wiklander, O. P. B. , Hällbrink, M. , Seow, Y. , Bultema, J. J. , Gilthorpe, J. , Davies, T. , Fairchild, P. J. , Gabrielsson, S. , Meisner‐Kober, N. C. , Lehtiö, J. , Smith, C. I. E. , Wood, M. J. A. , Andaloussi, S. E. L. , Nanomed. Nanotechnol. Biol. Med. 2015, 11, 879–883. 10.1016/j.nano.2015.01.003 25659648

[113] Baranyai, T. , Herczeg, K. , Onódi, Z. , Voszka, I. , Módos, K. , Marton, N. , Nagy, G. , Mäger, I. , Wood, M. J. , El Andaloussi, S. , Pálinkás, Z. , Kumar, V. , Nagy, P. , Kittel, Á. , Buzás, E. I. , Ferdinandy, P. , Giricz, Z. , PLoS One 2015, 10, e0145686. 26690353 10.1371/journal.pone.0145686 PMC4686892

[114] Bruce, T. F. , Slonecki, T. J. , Wang, L. , Huang, S. , Powell, R. R. , Marcus, R. K. , Electrophoresis 2019, 40, 571–581. 30548636 10.1002/elps.201800417 PMC6881775

[115] Lewis, G. D. , Metcalf, T. G. , Appl. Environ. Microbiol. 1988, 54, 1983–1988. 2845860 10.1128/aem.54.8.1983-1988.1988 PMC202790

[116] Martins, T. S. , Catita, J. , Rosa, I. M. , Da Cruz e Silva, O. A. B. , Henriques, A. G. , PLoS One 2018, 13, 1–17. 10.1371/journal.pone.0198820 PMC5995457 29889903

[117] Deregibus, M. C. , Figliolini, F. , D'Antico, S. , Manzini, P. M. , Pasquino, C. , De Lena, M. , Tetta, C. , Brizzi, M. F. , Camussi, G. , Int. J. Mol. Med. 2016, 38, 1359–1366. 28025988 10.3892/ijmm.2016.2759 PMC5065305

[118] Brownlee, Z. , Lynn, K. D. , Thorpe, P. E. , Schroit, A. J. , J. Immunol. Meth. 2014, 407, 120–126. 10.1016/j.jim.2014.04.003 PMC4077054 24735771

[119] Colombet, J. , Robin, A. , Lavie, L. , Bettarel, Y. , Cauchie, H. M. , Sime‐Ngando, T. , J. Microbiol. Meth. 2007, 71, 212–219. 10.1016/j.mimet.2007.08.012 17897741

[120] Liu, W. , Hu, J. , Zhou, K. , Chen, F. , Wang, Z. , Liao, B. , Dai, Z. , Cao, Y. , Fan, J. , Zhou, J. , OncoTargets Ther. 2017, 10, 3843–3851. 10.2147/OTT.S140062 PMC5546809 28814883

[121] Caradec, J. , Kharmate, G. , Hosseini‐Beheshti, E. , Adomat, H. , Gleave, M. , Guns, E. , Clin. Biochem. 2014, 47, 1286–1292. 24956264 10.1016/j.clinbiochem.2014.06.011

[122] Malla, B. , Aebersold, D. M. , Dal Pra, A. , J. Transl. Med. 2018, 16, 223. 30103771 10.1186/s12967-018-1592-6 PMC6090775

[123] Alvarez, M. L. , Khosroheidari, M. , Kanchi Ravi, R. , Distefano, J. K. , Kidney Int. 2012, 82, 1024–1032. 22785172 10.1038/ki.2012.256

[124] Rekker, K. , Saare, M. , Roost, A. M. , Kubo, A. L. , Zarovni, N. , Chiesi, A. , Salumets, A. , Peters, M. , Clin. Biochem. 2014, 47, 135–138. 24183884 10.1016/j.clinbiochem.2013.10.020

[125] Zhao, L. , Yu, J. , Wang, J. , Li, H. , Che, J. , Cao, B. , J. Cancer 2017, 8, 1145–1152. 28607588 10.7150/jca.18026 PMC5463428

[126] Zarovni, N. , Corrado, A. , Guazzi, P. , Zocco, D. , Lari, E. , Radano, G. , Muhhina, J. , Fondelli, C. , Gavrilova, J. , Chiesi, A. , Methods 2015, 87, 46–58. 26044649 10.1016/j.ymeth.2015.05.028

[127] Alvarez, M. L. , Methods Mol. Biol. 2014, 1182, 145–170. 25055908 10.1007/978-1-4939-1062-5_13

[128] György, B. , Módos, K. , Pállinger, É. , Pálóczi, K. , Pásztói, M. , Misják, P. , Deli, M. A. , Sipos, Á. , Szalai, A. , Voszka, I. , Polgár, A. , Tóth, K. , Csete, M. , Nagy, G. , Gay, S. , Falus, A. , Kittel, Á. , Buzás, E. I. , Blood 2011, 117, e39. 21041717 10.1182/blood-2010-09-307595

[129] Kalra, H. , Adda, C. G. , Liem, M. , Ang, C. S. , Mechler, A. , Simpson, R. J. , Hulett, M. D. , Mathivanan, S. , Proteomics 2013, 13, 3354–3364. 24115447 10.1002/pmic.201300282

[130] Chang, M. , Chang, Y.‐J. , Chao, P. Y. , Yu, Q. , PLoS One 2018, 13, e0199438. 29933408 10.1371/journal.pone.0199438 PMC6014651

[131] Ciardiello, C. , Cavallini, L. , Spinelli, C. , Yang, J. , Reis‐Sobreiro, M. , de Candia, P. , Minciacchi, V. R. , Di Vizio, D. , Int. J. Mol. Sci. 2016, 17, 175. 26861306 10.3390/ijms17020175 PMC4783909

[132] Hu, G. , Drescher, K. M. , Chen, X. M. , Front. Genet. 2012, 3, 56. 22529849 10.3389/fgene.2012.00056 PMC3330238

[133] Purushothaman, A. , Bandari, S. K. , Liu, J. , Mobley, J. A. , Brown, E. E. , Sanderson, R. D. , J. Biol. Chem. 2016, 291, 1652–1663. 26601950 10.1074/jbc.M115.686295 PMC4722448

[134] Samsonov, R. , Shtam, T. , Burdakov, V. , Glotov, A. , Tsyrlina, E. , Berstein, L. , Nosov, A. , Evtushenko, V. , Filatov, M. , Malek, A. , Prostate 2016, 76, 68–79. 26417675 10.1002/pros.23101

[135] Ueda, K. , Ishikawa, N. , Tatsuguchi, A. , Saichi, N. , Fujii, R. , Nakagawa, H. , Sci. Rep. 2014, 4, 6232. 25167841 10.1038/srep06232 PMC4148700

[136] Rabinowits, G. , Gerçel‐Taylor, C. , Day, J. M. , Taylor, D. D. , Kloecker, G. H. , Clin. Lung Cancer 2009, 10, 42–46. 19289371 10.3816/CLC.2009.n.006

[137] Sharma, P. , Ludwig, S. , Muller, L. , Hong, C. S. , Kirkwood, J. M. , Ferrone, S. , Whiteside, T. L. , J. Extracell. Vesicles 2018, 7, 1435138. 29511460 10.1080/20013078.2018.1435138 PMC5827723

[138] Brett, S. I. , Lucien, F. , Guo, C. , Williams, K. C. , Kim, Y. , Durfee, P. N. , Brinker, C. J. , Chin, J. I. , Yang, J. , Leong, H. S. , Prostate 2017, 77, 1335–1343. 28762517 10.1002/pros.23393

[139] He, F. , Liu, H. , Guo, X. , Yin, B. C. , Ye, B. C. , Anal. Chem. 2017, 89, 12968–12975. 29139297 10.1021/acs.analchem.7b03919

[140] Cai, S. , Luo, B. , Jiang, P. , Zhou, X. , Lan, F. , Yi, Q. , Wu, Y. , Nanoscale 2018, 10, 14280–14289. 30014056 10.1039/c8nr02871k

[141] Nakai, W. , Yoshida, T. , Diez, D. , Miyatake, Y. , Nishibu, T. , Imawaka, N. , Naruse, K. , Sadamura, Y. , Hanayama, R. , Sci. Rep. 2016, 6, 33935. 27659060 10.1038/srep33935 PMC5034288

[142] Liga, A. , Vliegenthart, A. D. B. , Oosthuyzen, W. , Dear, J. W. , Kersaudy‐Kerhoas, M. , Lab Chip 2015, 15, 2388–2394. 25940789 10.1039/c5lc00240k

[143] Kibria, G. , Ramos, E. K. , Lee, K. E. , Bedoyan, S. , Huang, S. , Samaeekia, R. , Athman, J. J. , Harding, C. V. , Lï¿½tvall, J. , Harris, L. , Thompson, C. L. , Liu, H. , Sci. Rep. 2016, 6, 36502. 27819324 10.1038/srep36502 PMC5098148

[144] Davies, R. T. , Kim, J. , Jang, S. C. , Choi, E. J. , Gho, Y. S. , Park, J. , Lab Chip 12, 2012, 5202–5210. 23111789 10.1039/c2lc41006k

[145] Xu, H. , Liao, C. , Zuo, P. , Liu, Z. , Ye, B. C. , Anal. Chem. 2018, 90, 13451–13458. 30234974 10.1021/acs.analchem.8b03272

[146] Shi, L. , Rana, A. , Esfandiari, L. , Sci. Rep. 2018, 8, 6751. 29712935 10.1038/s41598-018-25026-2 PMC5928082

[147] He, M. , Crow, J. , Roth, M. , Zeng, Y. , Godwin, A. K. , Lab Chip 2014, 14, 3773–3780. 25099143 10.1039/c4lc00662c PMC4161194

[148] Chiriacò, M. S. , Bianco, M. , Nigro, A. , Primiceri, E. , Ferrara, F. , Romano, A. , Quattrini, A. , Furlan, R. , Arima, V. , Maruccio, G. , Sensors 2018, 18, 3175. 10.3390/s18103175 PMC6210978 30241303

[149] Kanwar, S. S. , Dunlay, C. J. , Simeone, D. M. , Nagrath, S. , Lab Chip 2014, 14, 1891–1900. 24722878 10.1039/c4lc00136b PMC4134440

[150] Chen, C. , Skog, J. , Hsu, C. H. , Lessard, R. T. , Balaj, L. , Wurdinger, T. , Carter, B. S. , Breakefield, X. O. , Toner, M. , Irimia, D. , Lab Chip 2010, 10, 505–511. 20126692 10.1039/b916199f PMC3136803

[151] Wang, Z. , Wu, H. J. , Fine, D. , Schmulen, J. , Hu, Y. , Godin, B. , Zhang, J. X. J. , Liu, X. , Lab Chip 2013, 13, 2879–2882. 23743667 10.1039/c3lc41343h PMC3740541

[152] Gholizadeh, S. , Shehata Draz, M. , Zarghooni, M. , Sanati‐Nezhad, A. , Ghavami, S. , Shafiee, H. , Akbari, M. , Biosens. Bioelectron. 2017, 91, 588–605. 28088752 10.1016/j.bios.2016.12.062 PMC5323331

[153] Beit‐Yannai, E. , Tabak, S. , Stamer, W. D. , J. Cell. Mol. Med. 2018, 22, 2001–2006. 29377463 10.1111/jcmm.13479 PMC5824382

[154] Kowal, J. , Tkach, M. , Théry, C. , Curr. Opin. Cell Biol. 2014, 29, 116–125. 24959705 10.1016/j.ceb.2014.05.004

[155] Vlassov, A. V , Magdaleno, S. , Setterquist, R. , Conrad, R. , Biochim. Biophys. Acta, Gen. Subj. 2012, 1820, 940–948. 10.1016/j.bbagen.2012.03.017 22503788

[156] Wu, Y. , Deng, W. , Klinke, D. J. II , Analyst 2015, 140, 6631–6642. 26332016 10.1039/c5an00688k PMC4986832

[157] Jan, A. , Rahman, S. , Khan, S. , Tasduq, S. , Choi, I. , Cells 2019, 8, 99. 10.3390/cells8020099 PMC6406279 30699987

[158] Van Deun, J. , Mestdagh, P. , Sormunen, R. , Cocquyt, V. , Vermaelen, K. , Vandesompele, J. , Bracke, M. , De Wever, O. , Hendrix, A. , J. Extracell. Vesicles 2014, 3, 24858. 10.3402/jev.v3.24858 PMC4169610 25317274

[159] Sanz‐Rubio, D. , Martin‐Burriel, I. , Gil, A. , Cubero, P. , Forner, M. , Khalyfa, A. , Marin, J. M. , Sci. Rep. 2018, 8, 10306. 29985466 10.1038/s41598-018-28748-5 PMC6037782

